# Clinical Characteristics and CT Findings in 148 Non-COVID-19 Influenza-Like Illness Cases: A Retrospective Control Study

**DOI:** 10.3389/fpubh.2021.616963

**Published:** 2021-02-09

**Authors:** Weizheng Shuai, Xuxin Chen, Yi Shan, Wenping Li, Wei Ma, Qiaohui Lu, Dawei Li

**Affiliations:** ^1^Department of Critical Care Medicine, The Sixth Medical Center, Chinese PLA General Hospital, Beijing, China; ^2^Department of Respiratory and Critical Care Medicine, The Eighth Medical Center, Chinese PLA General Hospital, Beijing, China; ^3^Department of Emergency Medicine, The Sixth Medical Center, Chinese PLA General Hospital, Beijing, China; ^4^Radiology Department, The Sixth Medical Center, Chinese PLA General Hospital, Beijing, China; ^5^Basic Medical Research Center, The Sixth Medical Center, Chinese PLA General Hospital, Beijing, China

**Keywords:** computerized tomography, influenza-like illness, influenza, respiratory tract infection, ground glass opacities, consolidation

## Abstract

**Background:** This study was to collect clinical features and computed tomography (CT) findings of Influenza-Like Illness (ILI) cases, and to evaluate the correlation between clinical data and the abnormal chest CT in patients with the Influenza-Like Illness symptoms.

**Methods:** Patients with the Influenza-Like Illness symptoms who attended the emergency department of The Six Medical Center of The PLA General Hospital from February 10 to April 1, 2020 were enrolled. Clinical and imaging data of the enrolled patients were collected and analyzed. The association between clinical characteristics and abnormal chest CT was also analyzed.

**Results:** A total of 148 cases were enrolled in this study. Abnormalities on chest CT were detected in 61/148 (41.2%) patients. The most common abnormal CT features were as follows: patchy consolidation 22/61(36.1%), ground-glass opacities 21/61(34.4%), multifocal consolidations 17/61(27.9%). The advanced age and underlying diseases were significantly associated with abnormal chest CT.

**Conclusions:** Abnormal chest CT is a common condition in Influenza-Like Illness cases. The presence of advanced age and concurrent underlying diseases is significantly associated with abnormal chest CT findings in patients with ILI symptoms. The chest CT characteristic of ILI is different from the manifestation of COVID-19 infection, which is helpful for differential diagnosis.

## Introduction

Seasonal influenza is a global respiratory infectious disease (1). According to the statistics, the incidence of symptomatic seasonal influenza in the general population of the United States was approximately 8% between 2010 and 2016 (2). The disease is usually self-limiting, but it can also cause serious complications and even death in particular populations (3–9).

Based on the statistics, the number of hospitalizations associated with seasonal influenza in the United States is between 140,000 and 960,000 each year (7, 10). The global annual number of deaths caused by respiratory complications related to seasonal influenza is between 290,000 and 640,000 (3). Flu-like symptoms are usually present in such patients when they visit doctor. If the identification of patients in whom respiratory complications have occurred can be achieved as early as possible, the adverse outcomes of delayed diagnosis may be avoided.

Chest CT is an important examination in the diagnosis of lower respiratory tract infections. However, chest imaging, especially chest CT examination, is not usually conducted for the diagnosis and treatment of patients with influenza-like illness (1). Furthermore, existing studies tend to target patients with more serious illness, and there is a paucity of data related to overall lung imaging in influenza-like cases (11–15). Therefore, a general survey of lung imaging is helpful for recognizing the overall condition in the lung imaging of influenza-like cases and for providing a reference for clinical practice.

During the COVID-19 epidemic, in order to screen for patients with respiratory infections, chest CT was used extensively to examine patients with fever at our hospital, which provided us with an opportunity to know about the general characteristics of lung imaging in influenza-like cases. In the COVID-19 epidemic season at the beginning of 2020, we collected and analyzed chest imaging data on influenza-like cases (non-COVID-19) in a non-epidemic area (Ganjiakou area, Haidian District, Beijing, China), to provide a relevant reference for future imaging studies of influenza-like cases. Since lung imaging is an important element in the diagnosis of pneumonia, it plays a significant role in the selection of treatment plans. In this study, influenza-like cases were divided into a normal CT group and an abnormal CT group based on the results of chest CT examination, and a comparison between the groups as well as logistic regression analysis was performed to determine the relevant features with regards to lung imaging abnormalities.

## Materials and Methods

### Patients

Inclusion criteria: Patients who attended the emergency department of The Sixth Medical Center of Chinese PLA General Hospital because of respiratory infection symptom (RTI) from the 10th of February to the 1st April, 2020 were enrolled in this retrospective control study. RTI was defined as one with respiratory tract symptoms (i.e., new onset cough, sore throat, running nose or sputum production) (16).

**Figure 1:**
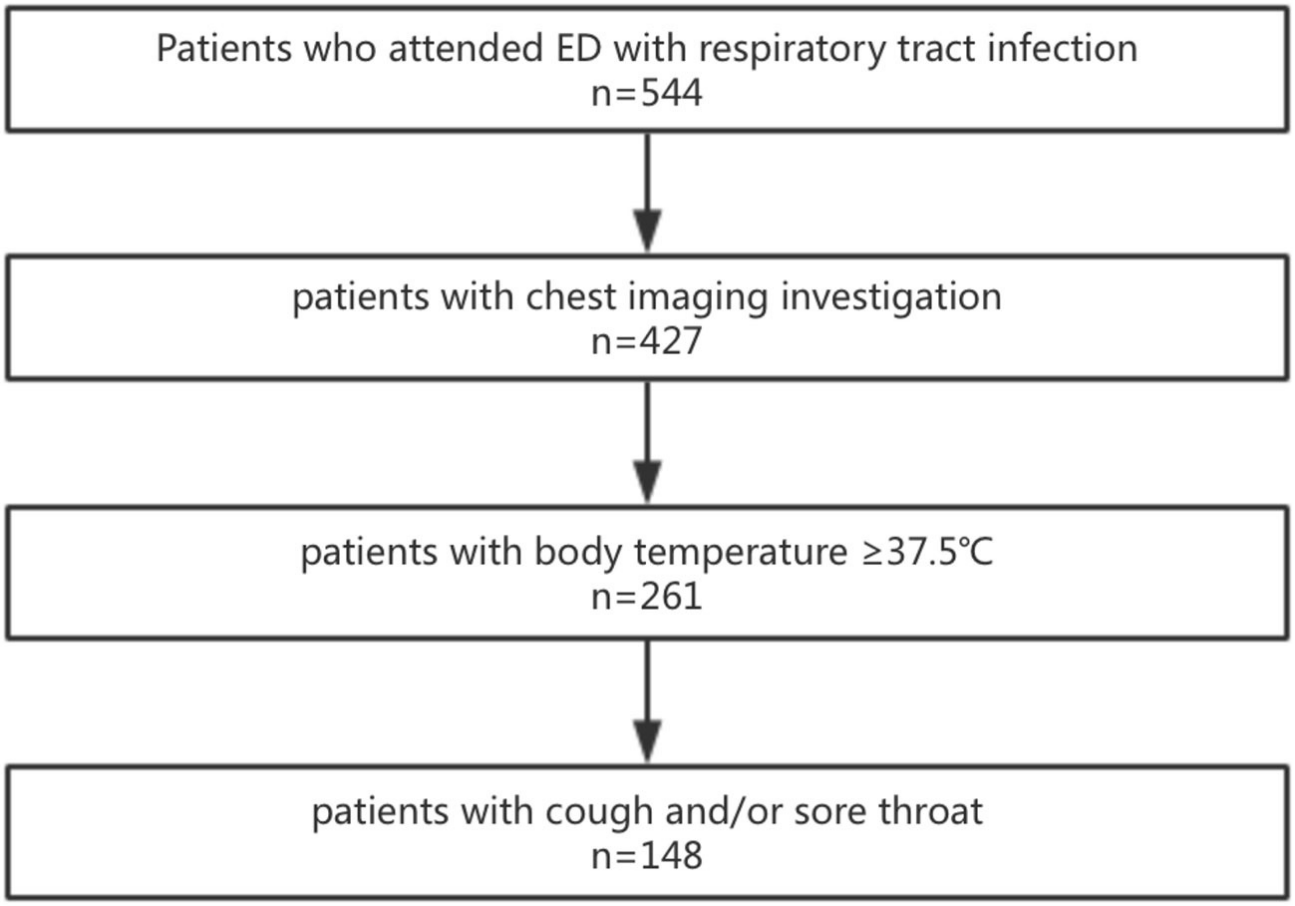
Flowchart of patient selection. 544 patients visited the emergency department with respiratory infection symptoms. 427 patients underwent the chest CT imaging examination. 261 patients with the body temperature ≥37.5°C. 148 patients with the symptoms of cough and/or sore throat.

Exclusion criteria: patients younger than 14 years old; patients without infection; patients with COVID-19 infection; cases without CT examination; patients who did not meet the criteria for ILI (body temperature ≥ 37.5°C, accompanied by cough and/ or sore throat) (17, 18). The axillary temperature of all patients were measured using the mercury meter, and the recorded body temperature was the highest value of the body temperature self-reported or measured in the hospital. Figure 1 showed flowchart of patient selection.

A diagnosis of COVID-19 was excluded in all enrolled patients during this period. Once the patients were suspected of COVID-19, they were first checked by the expert team at our hospital, and respiratory tract specimens were obtained and sent for RT-PCR testing. If the test was positive, a diagnosis of COVID-19 infection was established. If two tests were negative, the diagnosis was excluded.

Standardized data collection forms were used to extract related data such as epidemiological data, demographic data, clinical characteristics, laboratory test results etc. from electronic medical records. The specific data that were collected included the time of consultation, demographic data (gender, age), clinical symptoms, blood test results (routine blood test, C-reactive protein), and comorbidity records. All data were reviewed by two doctors (SWZ and CXX). If there was a difference in interpretation between the two, a third researcher (LDW) acted as an arbiter.

This retrospective study was approved by the Ethics Commission of the Sixth Medical Center of the PLA General Hospital (AF/SC-09/02.1) and the requirement for informed consent was waived by the Ethics Commission.

### Chest Imaging Studies

Scanning position: supine position, CT scan range was from the supraclavicular region to the adrenal glands. Philips Brilliance iCT 256-slice CT machine was used, with the following scanning parameters: collimation 128 × 0.625 mm, reconstruction slice thickness 0.9 mm, slice interval 0.9 mm, matrix 512 × 512; tube voltage 100 kVp [for scanning patients with body mass index (BMI) ≤24 kg/m^2^] or 120 kVp (BMI > 24 kg/m^2^); X-ray tube rotation speed 0.5 s/turn, pitch 0.992, automatic mAs technology (Dose Right; Philips Healthcare). Iterative model reconstruction (IMR) was adopted for reconstruction of all data. Display window: lung window, window level L-650 HU, window width W1700 HU; mediastinal window, window level L40 HU, window width W400 HU.

For patients undergoing chest CT examination, the radiological images were independently read by two certified radiologists and a report was made. The radiologists were aware that the patients had flu-like symptoms, but they did not know the clinical symptoms, signs, laboratory findings and past history of the patients.

The lung imaging abnormalities were analyzed based on their features, and the results were classified and summarized according to the characteristics below. The criteria for CT morphology were as follows: patchy consolidation, multifocal consolidation, ground-glass opacity, central lobular nodules, bronchial wall thickening, thickening of the interlobular septum, pleural fluid and other CT abnormalities, diagnosed in accordance with the definitions within the glossary of terms by Fleischner (19).

The difference between patchy consolidation and multifocal consolidation lies in the extent and number of lesions. Patchy consolidation lesions have a diameter >1 cm or a diameter which exceeds a single slice, while multifocal consolidation lesions have a diameter of <1 cm, with three or more lesions present.

A normal appearance or pre-existing lung disease (such as emphysema, bronchiectasis, interstitial lung disease, tumor, old tuberculous foci etc.) without new lesions were defined as non-pathological findings. In addition, the distribution of anatomical lesions was classified into whole lung, upper, middle, or lower lung depending on the main area of the lesion. The location of the lesion was classified as segmental, lobular or diffuse distribution, or random distribution.

### Statistics

Continuous and categorical variables were presented as median (IQR) and *n* (%), respectively. SPSS for Windows software package, version 20.0 (SPSS Inc, Chicago, IL) was used. Mann-Whitney *U* test or χ^2^ test was used to compare differences between the normal CT group and the abnormal CT group. To explore the risk factors associated with abnormal CT findings, univariate and multivariate logistic regression models were used. By referring to the relevant literature, we selected age, gender, body temperature, underlying diseases, symptoms, white blood cell count, neutrophil count, lymphocyte count, and C-reactive protein as variables for stepwise logistic regression analysis (20). At the same time, parameters of odds ratios that were difficult to estimate were excluded due to the small number of cases as well as the parameters that had collinearity with the underlying disease. A *P*-value < 0.05 was considered statistically significant.

## Results

### Patients Characteristics

Through HIS system retrieval, a total of 544 adult patients with respiratory tract infection symptoms attended the emergency department of our hospital between February 10 and April 1, 2020, and among them, 117 patients did not undergo imaging investigation. Screening was performed based on the criteria of body temperature ≥37.5°C, and 166 patients with body temperature lower than 37.5°C were excluded. Based on at least one of the symptoms of cough or sore throat, 113 cases were further excluded, and 148 cases were finally included. At this stage, our hospital did not accept patients with COVID-19 infection (diagnosis was based on the diagnosis and treatment guidelines issued by the Ministry of Health of China) (21).

The demographic data and basic medical history of the patients are summarized in Table 1. Among the 148 patients who were finally included, the median age was 36 years (26, 58.75), of which 72 were males (48.6%). A total of 23 patients had underlying diseases, including diabetes, tuberculosis, COPD, cerebral stroke sequelae, fractures, bronchiectasis, chronic kidney disease, thyroid disease, hypertension, coronary heart disease, hyperlipidemia, solid tumors, Alzheimer's disease, hematological disease, autoimmune disease etc. In the abnormal imaging group, the median age was 66 years (38.5, 87), and 22 cases (36.1%) had underlying diseases. These two indicators were significantly higher than those of the normal group (*P* < 0.01).

**Table 1:** Patients demographic and basic medical history data.

	**Total (*n* = 148)**	**Normal (*n* = 87)**	**Abnormal (*n* = 61)**	***P*-value**
Sex				0.576
Male	72 (48.6%)	44 (50.6%)	28 (45.9%)	
Female	76 (51.4%)	43 (49.4%)	33 (54.1%)	
Age	36 (26,58.75)	28 (23,38)	66 (38.5,87)	0.000
Underling disease	23 (15.5%)	1 (1.1%)	22 (36.1%)	0.000
Diabetes	1 (0.7%)	0 (0%)	1 (1.6%)	0.231
Tuberculosis	1 (0.7%)	0 (0%)	1 (1.6%)	0.231
COPD	4 (2.7%)	0 (0%)	4 (6.6%)	0.015
Cerebral stroke sequelae	5 (3.4%)	0 (0%)	5 (8.2%)	0.07
Fracture	1 (0.7%)	0 (0%)	1 (1.6%)	0.231
Bronchiectasis	2 (1.4%)	1 (1.1%)	1 (1.6%)	0.799
Chronic kidney disease	3 (2.0%)	0 (0%)	3 (4.9%)	0.037
Thyroid disease	1 (0.7%)	0 (0%)	1 (1.6%)	0.231
Hypertension	2 (1.4%)	0 (0%)	2 (3.3%)	0.089
Coronary heart disease	0 (0%)	0 (0%)	0 (0%)	
Hyperlipidemia	1 (0.7%)	0 (0%)	1 (1.6%)	0.231
Solid tumors	1 (0.7%)	0 (0%)	1 (1.6%)	0.231
Alzheimer's disease	2 (1.4%)	0 (0%)	2 (3.3%)	0.089
Hematological disease	1 (0.7%)	0 (0%)	1 (1.6%)	0.231
Autoimmune disease	1 (0.7%)	0 (0%)	1 (1.6%)	0.231

*Data are median (IQR), n (%), or n/N (%). p-values were calculated by Mann-Whitney U test or χ^2^ test as appropriate. COPD, chronic obstructive pulmonary disease*.

### Imaging Analysis

**Figure 2:**
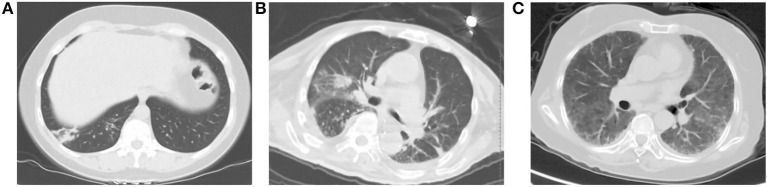
Patients with abnormal CT findings. **(A)** A 43-year-old woman presenting with fever, cough and expectoration. An unenhanced chest CT image shows patchy consolidation in right lower lobe. **(B)** A 85-year-old man presenting with high fever, cough and short of breath. An unenhanced chest CT image shows multifocal areas of consolidation in the bilateral lungs. **(C)** A 73-year-old woman presenting with fever and short of breath. She was diagnosed with Sjogren's syndrome 6 months ago, and she was receiving steroid drugs. An unenhanced chest CT examination shows ground-glass opacity widely scattered across the lungs bilaterally.

Among the included patients, a total of 61 patients had imaging abnormalities (41.2%). The chest CT findings are summarized in Table 2. Among them, there were 22 cases (36.1%) with multifocal consolidations, 21 (34.4%) cases with ground glass lesions, and 17 cases (27.9%) with patchy consolidations. There were 12 (19.7%) cases with bronchial wall thickening, and 8 (13.1%) with centrilobular nodules. There were three cases (4.9%) with interlobular thickening and two cases (3.3%) with lymphadenopathy. Among 61 patients, seven cases (11.48%) had pleural effusion, of which one (1.64%) had bilateral pleural effusion, and six (9.84%) had unilateral pleural effusion. Figure 2 showed patients with abnormal CT findings.

**Table 2:** CT characteristics in 61 abnormal CT findings.

**Abnromal CT findings**	**Cases (*n* = 61)**	**Proportion (%)**
Patchy consolidations	17	27.9
Multifocal consolidations	22	36.1
Ground-glass opacities	21	34.4
Centrilobular nodules	8	13.1
Bronchial wall thickening	12	19.7
Interlobular septal thickening	3	4.9
Lymphadenopathy	2	3.3
Pleural effusion	7	11.5
Unilateral	6	9.8
Bilateral	1	1.6
**Anatomical distribution zonal predominance**		
Upper and mid	18	29.5
Lower	27	44.3
Whole lung	16	26.23
**Localization**		
Segmental and lobar	49	80.33
Diffuse	12	19.67
**Symmetry**		
Bilateral	30	49.2
Unilateral	31	50.8

*CT, computed tomography*.

Based on the statistics related to the distribution characteristics of lesions, it can be seen that there were 31 cases (50.8%) with unilateral lesions, and 30 patients (49.2%) with bilateral lesions. In terms of classification based on the distribution area of the main lesions, there were 18 (29.5%) in the upper lung, 27 (44.3%) in the lower lung, and concurrent involvement of the upper, middle, and lower lung was present in 16 (26.2%). Based on lesion location classification, 49 (80.3%) of the main lesions were distributed along the lobes/segments of the lung, with fewer cases of diffuse distribution (12, 19.7%).

### Symptom Analysis

The clinical characteristics of the 148 patients in this study are summarized in Table 3. The patient's body temperature was 37.9°C (37.5, 40.2°C) at the time of consultation. The most common clinical symptoms were sore throat 79/148 (53.7%), cough 86/148 (58.1%), and sputum 44/148 (29.7%), followed by fatigue and shortness of breath. Vomiting and diarrhea were relatively rare.

**Table 3:** Symptom and laboratory results.

**Symptom**	**Total (*n* = 148)**	**Normal (*n* = 87)**	**Abnormal (*n* = 61)**	***P*-value**
Body temperature	37.9 (37.6, 38.5)	37.9 (37.6, 38.4)	38.0 (37.6, 38.55)	0.26
Cough	86 (58.1%)	39 (44.8%)	47 (77.0%)	0.000
Sore throat	79 (53.7%)	58 (66.7%)	21 (34.4%)	0.000
Sputum	44 (29.7%)	18 (20.7%)	26 (42.6%)	0.004
Rhinocleisis	9 (6.1%)	6 (6.9%)	3 (4.9%)	0.620
Running nose	20 (13.5%)	15 (17.2%)	5 (8.2%)	0.113
Sneeze	5 (3.4%)	3 (3.4%)	2 (3.2%)	0.955
Headache	14 (9.5%)	11 (12.6%)	3 (4.9%)	0.114
Myalgia	16 (10.8%)	12 (13.8%)	4 (6.6%)	0.163
Fatigue	28 (18.9%)	22 (25.3%)	6 (9.8%)	0.018
Chill	17 (11.5%)	11 (12.6%)	6 (9.8%)	0.598
Diarrhea	3 (2.0%)	2 (2.3%)	1 (1.6%)	0.779
Vomiting	3 (2.0%)	2 (2.3%)	1 (1.6%)	0.779
Chest pain	4 (2.7%)	3 (3.4%)	1 (1.6%)	0.504
Shortness of breath	22 (14.9%)	9 (10.3%)	13 (21.3%)	0.065
**Laboratory results**				
Leucocyte^a^	8.23 (6.45, 11.52)	8.05 (6.65, 10.98)	8.32 (6.32, 11.92)	0.992
Neutrophil^a^	5.83 (4.12, 9.1)	5.79 (3.93, 8.91)	5.84 (4.27, 9.68)	0.67
Leukomonocyte^a^	1.37 (1.07, 1.96)	1.4 (1.10, 1.98)	1.29 (1.0, 1.93)	0.369
C reactive protein^b^	9.95 (1.33, 45.58)	5.4 (0.73, 27.15)	19.75 (4.23, 66.20)	0.005

*Data are median (IQR), n (%), or n/N (%). p-values were calculated by Mann-Whitney U test or χ^2^ test as appropriate*.

a *One hundred forty seven patients underwent complete blood cell count tests*.

b *One hundred forty four patients received C reactive protein examination*.

### Laboratory Examination Results

In this study, 147 patients underwent routine blood test and 144 patients accepted C-reactive protein test. The results showed that lymphopenia accounted for 27.2%, leukocytosis accounted for 39.5%, and leukopenia accounted for only 1.4%. More than half of the patients had elevated C-reactive protein levels.

In univariable analysis, patients with underlying diseases, cough and sputum, had a higher odds ratio of abnormal CT findings. In addition, fatigue, sore throat, advanced age, and elevated C reactive protein were also associated with abnormal chest CT findings (Table 4).

**Table 4:** Risk factors associated with chest CT findings.

	**OR**	**95% CI**	***P*-value**	**OR**	**95% CI**	***P*-value**
Age	1.070	(1.046–1.094)	0.000	1.059	(1.034-1.085)	0.000
Body temperature	1.350	(0.837–2.178)	0.219			
Underling disease	48.513	(6.312–372.877)	0.000	9.379	(1.069–82.249)	0.043
Cough	4.132	(1.989–8.584)	0.000			
Sputum	2.848	(1.378–5.883)	0.005			
Sore throat	0.263	(0.132–0.524)	0.000			
Shortness of breath	2.347	(0.933–5.907)	0.070			
Rhinocleisis	0.698	(0.168–2.907)	0.622			
Running nose	0.429	(0.147–1.250)	0.121			
Sneeze	0.949	(0.154–5.857)	0.955			
Headache	0.357	(0.095–1.340)	0.127			
Myalgia	0.439	(0.134–1.431)	0.172			
Fatigue	0.322	(0.122–0.852)	0.022			
Chill	0.754	(0.263–2.161)	0.599			
Diarrhea	0.708	(0.063–7.991)	0.780			
Vomiting	0.708	(0.063–7.991)	0.780			
Chest pain	0.467	(0.047–4.596)	0.514			
Leukocytosis^a^	0.882	(0.45–1.729)	0.715			
Neutrophilia^b^	0.957	(0.495–1.851)	0.896			
Lymphopenia^c^	0.714	(0.344–1.485)	0.367			
Elevated CRP^d^	2.622	(1.283–5.357)	0.008			

a Leukocyte > 9.5 × 10^9^/L;

b neutrophil > 6.3 × 10^9^/L;

c Lymphocyte < 1.1 × 10^9^/L;

d *C reactive protein > 5 mg/L*.

In the multivariable logistic regression model, all the variables of 144 patients were included. The results showed that advanced age and underlying disease were related to abnormal chest CT findings (Table 4).

## Discussion

As we known, ILI is a symptom complex which is a predictive tool for influenza infection. Compared with other clinical definitions, the commonly used definition of ILI is relatively broad to capture influenza-associated illness (22). It is often used as a key indicator for influenza surveillance. However, there is a paucity of data related to chest CT in influenza-like illness patients. We undertook this study to better understand the overall condition in the chest CT examination of ILI cases which may bring new knowledge to the area.

The current study analyzed the influenza-like cases that attended our hospital in the spring of 2020. The demographic characteristics, clinical symptoms, laboratory results, comorbidities, and chest CT imaging data of the enrolled patients were collected. The comparison of relevant indicators as well as logistic regression analysis were conducted on patients with normal chest CT and abnormal chest CT. In this study, the incidence of chest CT abnormalities, the main imaging features as well as the distribution pattern in influenza-like cases were analyzed. In addition, logistic regression analysis was used to identify risk factors associated with abnormal chest CT findings.

After conducting an epidemiological history as well as a survey of symptoms, signs, imaging, and differential diagnoses which included the use of nucleic acid test in accordance with the clinical diagnosis and treatment guidelines in China, COVID-19 infection was excluded in the enrolled cases. Therefore, the data reflected the relevant characteristics of non-COVID-19 influenza-like cases who attended hospital during this period.

In this study, 78.5% (427/544) of patients who presented with the main manifestations of respiratory symptoms underwent chest CT examination. According to our literature search, there have been no specific chest CT imaging studies on ILI cases, and to our knowledge, this study also enrolled the largest coverage of chest CT data in RTI patients (11, 13, 14). There were a few clinical reports about a varies of respiratory infection patients. However, in these studies, the proportion of patients with respiratory symptoms undergoing chest CT examinations did not exceed 20% and, furthermore, the patients were not randomized into groups (12, 16, 23–25). Whereas the limitations of previously research, this study conducted a comprehensive collection of chest CT data for patients with influenza-like symptoms and compared the difference of normal and abnormal chest CT cases. It may be noted that our research results can better reflect the overall characteristics of chest CT in ILI cases.

Among the 148 patients who met the diagnostic criteria for influenza-like cases, the proportion of chest CT abnormalities was 41.2%. Compared with the above-mentioned studies on lower respiratory tract infections involving specific pathogens, the proportion of chest CT positive cases was not high. However, after careful examination of these papers, it can be seen that the proportion of patients who underwent chest CT examination in the existing studies was too small, and thus they cannot reflect the general condition with regards to the group of influenza like illness patients (generally, clinicians only provided chest CT examinations when the condition was severe). Moreover, it can be seen from imaging analysis that abnormal chest CT phenomena in influenza-like cases is not uncommon. This can act as a reminder for clinicians that when treating patients with influenza-like symptoms, they should pay more attention to the changes in the pulmonary condition of patients, and chest CT examination should be performed more readily in high-risk groups.

In order to identify high-risk groups in influenza-like cases with abnormal lung imaging, logistic regression analysis was performed on the case data that were collected. From the results of univariable analysis, it can be seen that advanced age, underlying diseases, cough, sputum and increased CRP levels were risk factors for abnormal chest CT. Sore throat and symptoms of fatigue were associated with normal chest CT. With regards to an analysis of the cause, it may be that these two symptoms tend to appear in the early stages of disease onset, when the condition has not yet progressed to the lower respiratory tract.

After multivariable logistic regression analysis, it was found that advanced age and concurrent underlying diseases were independent risk factors for abnormal chest CT. These results are consistent with those of previous researchers.

A number of studies have suggested that as age increases, the body's immune function changes and the ability to resist pathogen invasion decreases. Saskia et al. found in animal experiments that the elderly macaques exhibited a higher level of systemic inflammatory response following coronavirus infection, but the synthesis and secretion of type I interferon β, which inhibits virus replication, was insufficient, which made it difficult to control the infection (26). It has also been found that the decline of immune function in the elderly is mainly featured by the gradual decrease of the naive CD8 T cell repertoire, and the decline in the ability of CD4 T cells to recognize foreign antigens and generate an immune response (27). As a result of the reduced capacity to resist pathogenic microorganisms such as viruses and bacteria, local infections easily spread to the whole body, leading to a rapid deterioration in the person's condition. This can also explain why the elderly are prone to developing lower respiratory tract infections following the occurrence of upper respiratory tract infections.

In addition, concurrent underlying disease is also a common risk factor of lung disease. Irene RC's research showed that the incidence of community-acquired pneumonia increased sharply with age, and was related to underlying conditions in the patient, particularly chronic obstructive pulmonary disease, asthma, chronic cardiovascular disease, impaired immune function, and neurological diseases (28). A number of recent meta-analyses of retrospective studies on COVID-19 confirmed that advanced age, COPD, hypertension, diabetes, cardiovascular disease and cerebrovascular disease are associated with a significantly increased risk of exacerbation in COVID-19 patients, among which the risk of disease progression in COPD patients is 5.9 times that of non-COPD patients (29, 30).

The results of the current study are also indicative of this feature. Among patients with pulmonary lesions, the incidence of underlying conditions was significantly higher than that of patients with normal lung imaging. However, due to the small number of single disease types within the comorbidities in this study (overall, <4%), single disease subgroup analysis was not performed.

Results of logistic regression analysis showed that the elderly patients with underlying diseases were more likely to develop lower respiratory tract inflammation following upper respiratory tract infection. This also provides a basis for clinicians to distinguish high-risk groups when receiving ILI cases. Therefore, particular attention needs to be paid to this group in clinical practice, and the timing of treatment also needs to be moved forward. However, these conjectures need to be demonstrated by prospective studies with larger sample sizes.

This study also conducted a corresponding analysis of abnormal chest CT findings between ILI cases and COVID-19 patients. According to a literature review, the imaging manifestations of lower respiratory tract infections caused by different pathogens are different. Taking influenza virus pneumonia as an example, the main abnormal CT characteristics are ground glass opacity and consolidation, as well as diffuse distribution in both lungs (16, 31, 32). Kim et al. found that patients with parainfluenza virus infection are more prone to tree-in-bud opacities and bronchial wall thickening (14). At the same time, such changes tend to be in the lower lung and distributed diffusely. Chest CT changes in patients with pneumonia caused by syncytial virus typically involve thickening of the bronchial wall, and lesions are mostly located in the upper and middle lung (16). In lower respiratory tract infections caused by bacteria, the most common chest CT abnormalities are consolidation and ground glass shadows, followed by bronchial wall thickening, and the proportion with a diffuse distribution of lesions is even higher than that of viral infections (16).

The imaging characteristics in this study were different from those in the above reports. Among the 61 cases with imaging changes, over half had chest CT consolidation, while the incidence of ground glass shadows was relatively low (22, 34.4%), and the lesions were more often located in the lower lungs and predominantly distributed in the pulmonary segments and lobes.

However, the performance of COVID-19 is different. In terms of CT features, COVID-19 is associated with ground glass lesions more often than consolidation, reaching as high as 98% (33)^.^ And, according to reports, the proportion of lung consolidation in the later stages of the disease gradually increases. With regards to distribution characteristics, the lesions are mostly located under the pleura or along the bronchovascular bundle (34–37).

In addition, according to the literature, the incidence of chest thickening and pleural effusion in non-COVID-19 patients is significantly higher than that of COVID-19 patients (36). However, there are inconsistent views on this issue in different literatures. Some scholars have found that pleural thickening but not accompanied by pleural effusion was often seen in chest CT of COVID-19 patients (38). In our study, the incidence of pleural effusion was low (7, 11.5%), and no pleural thickening was observed. The results suggest that, based only on the evidence of pleural effusion or pleural thickening can not effectively distinguish the two COVID-19 and non-COVID-19 cases, so further research is needed.

Once again, multiple studies have found that the halo and reverse halo signs often appear in COVID-19 patients, but these two signs are rarely seen in non-COVID-19 patients (36, 39–41). However, in the cases enrolled in this study, no halo sign and reverse halo sign was seen in the abnormal signs of chest CT. This result is consistent with previous reports. This means that the appearance of these two signs may indicate that patients are more likely to have COVID-19 infection, which can be used for the differential diagnosis of COVID-19.

Due to limited conditions, it was difficult to obtain respiratory tract samples from patients who attended the emergency department at our hospital, particularly samples of lower respiratory tract secretions, for nucleic acid testing and pathogen isolation and culture. Therefore, in this study, we did not have a pathogenic basis for performing an analysis of chest CT features based on etiology. However, it can still be seen from the current data that there are still significant differences in chest CT features between influenza-like patients without COVID-19 and patients with COVID-19 infection. CT can thus be used as a rapid screening tool for patients with suspected COVID-19.

There are limitations to this study. First of all, this was a single-center observational study which may not be representative of the general population. Secondly, it needs to be emphasized that influenza-like symptoms are not always caused by viral infections, and other pathogens such as bacteria and mycoplasma as well as non-infectious factors must necessarily be included. However, current data suggest that respiratory viruses are still the most important pathogenic microorganisms in this patient group (42). Moreover, when clinicians are conducting their day-to-day work, pathogen identification is not necessarily the main factor that influences the direction of diagnosis and treatment. Therefore, our results are still helpful for clinicians in their daily work. They can act as a reminder for doctors to focus more on the chest imaging of patients who are elderly and have concurrent underlying conditions. Thirdly, this study was conducted during the novel coronavirus epidemic and, due to precautionary considerations, patients with mild illness were more likely to choose to isolate at home and attend the hospital only when symptoms were more significant. This may have led to an overestimation of the occurrence of abnormal imaging in influenza-like cases. In addition, there were no patients with COVID-19 infection in this study, so direct comparisons could not be made. To overcome these limitations, we plan to conduct multi-center clinical study to address in the next phase.

Despite these limitations, we were able to comprehensively examine the chest CT features of ILI in this study. At the same time, we also believe that this work can prompt physicians to increase the attention they pay to non-COVID-19 ILI cases in clinical practice.

## Conclusions

1. A relatively high proportion of patients with influenza-like illness have chest CT abnormalities.
2. In influenza-like cases, advanced age and concurrent underlying diseases are related to abnormal chest CT findings, indicating that the risk of lower respiratory tract infection is higher in this patient group.
3. At present, the abnormal chest CT manifestations of influenza-like cases in Beijing urban areas are mainly lung consolidation, followed by ground glass lesions. Distinctive differences exist with regards to the distribution characteristics of imaging abnormalities compared to those associated with COVID-19 infection, which is helpful for differential diagnosis.

## Data Availability Statement

The original contributions presented in the study are included in the article/Supplementary Material, further inquiries can be directed to the corresponding author/s.

## Ethics Statement

The studies involving human participants were reviewed and approved by Ethics Commission of the Sixth Medical Center of the PLA General Hospital. Written informed consent from the participants' legal guardian/next of kin was not required to participate in this study in accordance with the national legislation and the institutional requirements.

## Author Contributions

WS, XC, YS, and DL conceived the study and participated in its design and coordination of the research. WS, XC, and YS contributed to the interpretation of the results. WS and WM developed, modeled, and performed evaluations and statistical analysis. WL and QL provided the radiological analyses and drew the CT conclusion. All authors have contributed to the drafting of the manuscript and have read and approved the final version.

## Conflict of Interest

The authors declare that the research was conducted in the absence of any commercial or financial relationships that could be construed as a potential conflict of interest.
